# Supplementary material for: Antibiotic resistance begets more resistance: chromosomal resistance mutations mitigate fitness costs conferred by multi-resistant clinical plasmids
Source: Microbiol Spectr. 2024 Mar 27;12(5):e04206-23. doi: 10.1128/spectrum.04206-23 (PMC11064507; doi:10.1128/spectrum.04206-23)
Supplement: Supplemental material — Tables S1 to S3 and Figures S1 to S3. [file spectrum.04206-23-s0001.pdf]

Supplementary information:

Antibiotic resistance begets more resistance: Chromosomal resistance mutations mitigate fitness costs conferred by multiresistant clinical plasmids

Ramith R. Nair*, Dan I. Andersson and Omar M. Warsi

Department of Medical Biochemistry and Microbiology, Uppsala University, Uppsala, SE-75123, Sweden

*Correspondence to: ramith_nair@hotmail.com

Table S1: **List of strains used in the study along with their description**

Strain Name	Description
DA5438	Wildtype strain of MG1655, sensitive to all antibiotics and the parent strain to all other strains used here
DA28100	DA5438 labelled with YFP
DA28102	DA5438 labelled with BFP
DA49842	DA5438 containing mutation S83L in the gene *gyrA* (CIP-R)
DA49828	DA5438 containing mutation K42N in the gene *rpsL* (STR-R)
DA65117	DA5438 containing deletion of genes *nfsA* and *nfsB* (NIT-R)
DA69649	DA65117 labelled with YFP (NIT-R)
DA69650	DA65117 labelled with BFP (NIT-R)

Table S2: ***t* test results for relative exponential growth rate.** Results after testing each average relative exponential growth rate against 1 using Welch's one-sample two-sided *t* tests for each plasmid (column 1) in each of the host backgrounds (top row). df represents degrees of freedom and p values represent the values after multiple testing corrections (FDR).

Plasmid	Susceptible			NIT-R			CIP-R			STR-R		
	t	df	p (corr)	*t*	df	p (corr)	*t*	df	p (corr)	*t*	df	p (corr)
P1	-1.356	4	0.16	0.14	4	0.529	8.044	4	0.008	0.151	4	0.887
P2	1.563	9	0.924	0.704	9	0.406	4.552	4	0.039	0.71	4	0.807
P3	-4.859	5	0.009	0.28	6	0.512	2.139	4	0.161	0.798	4	0.807
P4	-3.106	5	0.025	0.305	8	0.512	4.004	4	0.042	-0.56	4	0.807
P5	-2.93	5	0.026	3.631	3	0.107	1.998	4	0.168	1.368	4	0.807
P6	-5.842	5	0.007	0.804	11	0.406	1.2	4	0.385	0.207	4	0.887
P7	-3.997	4	0.021	-0.224	5	0.633	0.063	4	0.953	-0.727	4	0.807
P8	-3.106	5	0.025	1.994	5	0.167	0.887	5	0.492	-0.812	4	0.807
P9	-4.647	5	0.009	3.156	5	0.107	0.744	5	0.531	0.648	4	0.807
P10	1.378	5	0.924	0.962	4	0.406	3.211	5	0.044	-0.17	4	0.887
P11	-0.395	6	0.417	-0.393	5	0.645	3.848	5	0.039	-0.727	4	0.807
P14	-7.236	5	0.005	1.014	5	0.406	12.011	5	0.001	1.164	4	0.807
P15	-2.782	5	0.028	2.583	5	0.107	3.56	4	0.044	0.151	4	0.887

Table S3: **MIC of resistant strains carrying the appropriate plasmids.** Numbers represent the MIC of the respective antibiotic in mg/L. Each host is tested against the antibiotic for which it carries the mutation in the chromosome. Two different NIT-R hosts were used in the study. Rep1 and Rep2 indicates the two biological replicates.

**Plasmid**	**NIT-R (YFP)**		**NIT-R (BFP)**		**CIP-R**		**STR-R**	
	**Rep1**	**Rep2**	**Rep1**	**Rep2**	**Rep1**	**Rep2**	**Rep1**	**Rep2**
None	48	48	48	48	0.38	0.38	>1024	>1024
P1	32	32	32	48	0.38	0.38	>1024	>1024
P2	32	48	48	32	0.25	0.38	>1024	>1024
P3	48	32	48	48	2	2	>1024	>1024
P4	48	48	48	48	NA	NA	>1024	>1024
P5	48	48	32	48	NA	0.38	>1024	>1024
P6	32	48	32	48	0.25	0.25	>1024	>1024
P7	32	48	32	48	0.25	0.38	>1024	>1024
P8	32	48	48	48	0.38	0.38	>1024	>1024
P9	48	48	48	48	0.25	0.38	>1024	>1024
P10	48	48	32	32	0.16	0.38	>1024	>1024
P11	32	32	48	48	0.25	0.25	>1024	>1024
P14	48	48	32	32	0.25	0.25	>1024	>1024
P15	32	32	48	48	0.38	0.38	NA	NA

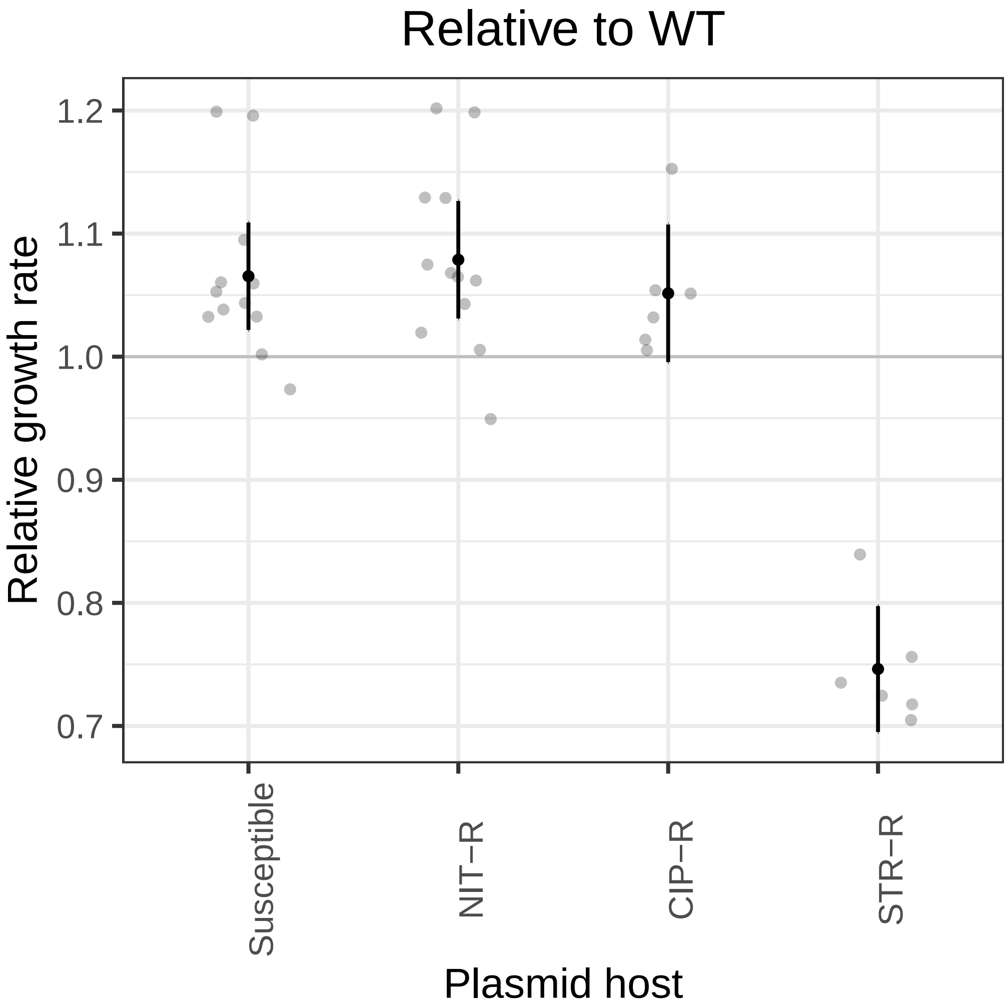

Figure S1: **Fitness effects of resistance mutations.** Relative exponential growth rates of the the four genetic backgrounds (Susceptible, NIT-R, CIP-R, and STR-R) relative to the wildtype MG155 in minimal glucose media. A value of 1 (horizontal line) represents no change in growth rate for plasmid-bearing strain when compared to the plasmid-free one, while a value less than one indicates that a fitness cost is conferred by the plasmid. Lighter points depict individual replicates, darker points represent means and error bars represent 95% confidence intervals (t-distribution, n = 6 or 12).

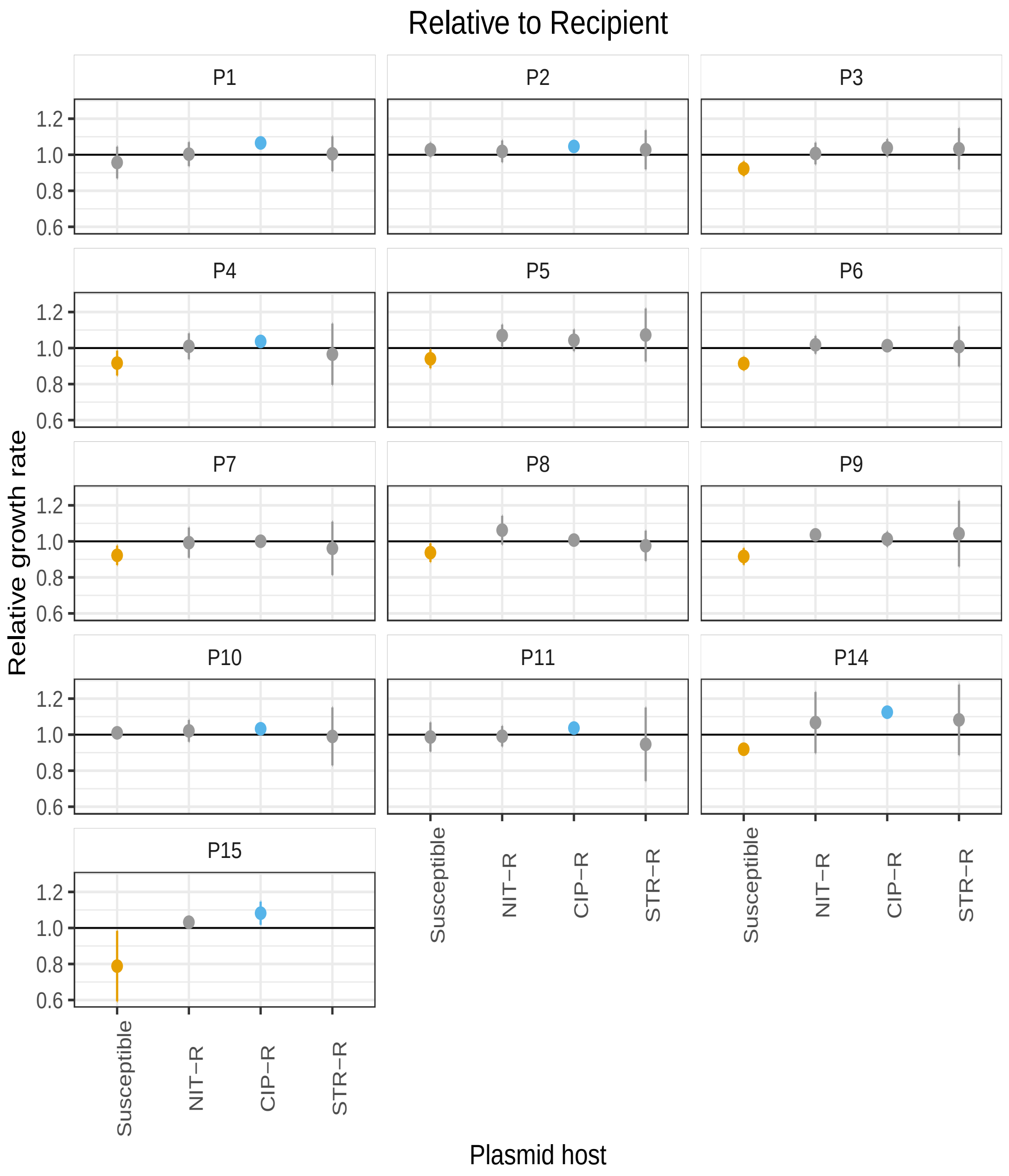

Figure S2: **Plasmids do not have a uniform effect across backgrounds.** Relative exponential growth rates in each of the the four genetic backgrounds (Susceptible, NIT-R, CIP-R, and STR-R) for each plasmid. Plasmid ID is mentioned as heading for each panel. A value of 1 (horizontal line) represents no change in growth rate for plasmid-bearing strain when compared to the plasmid-free one, while a value less than one indicates that a fitness cost is conferred by the plasmid. Orange squares depict relative exponential growth rates significantly lower than 1, blue triangles depict values higher than 1 and grey circles depict values equal to 1 (following Welch's two-sided one-sample t tests against 1). Points represent means and error bars represent 95% confidence intervals (t-distribution, n = 4 − 11 (Table S1)).

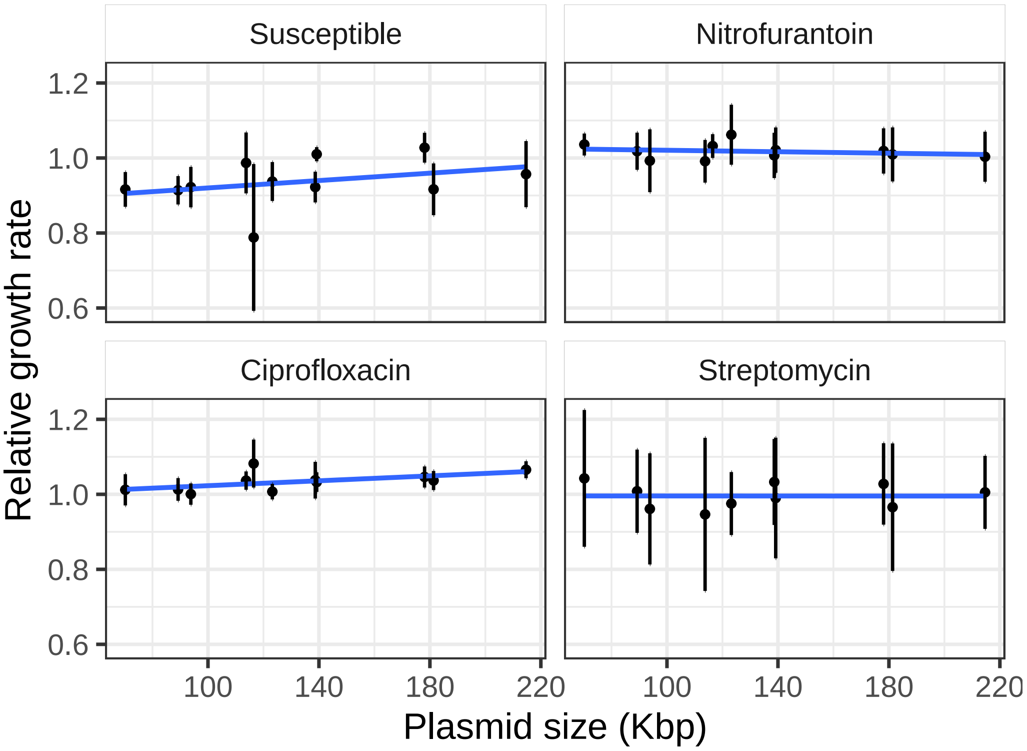

Figure S3: **Correlation between relative exponential growth rates and plasmid size.** Depiction of correlation between relative exponential growth rates and plasmid size across the four genetic backgrounds, (a) Susceptible, (b) NIT-R, (c) CIP-R , (d) STR-R.
